# Proteome analysis of soybean leaves, hypocotyls and roots under salt stress

**DOI:** 10.1186/1477-5956-8-19

**Published:** 2010-03-29

**Authors:** Hamid Sobhanian, Roya Razavizadeh, Yohei Nanjo, Ali Akbar Ehsanpour, Ferdous Rastgar Jazii, Nasrin Motamed, Setsuko Komatsu

**Affiliations:** 1 National Institute of Crop Science, Tsukuba 305-8518, Japan; 2 School of Biology, College of Science, University of Tehran, Tehran 14155-6455, Iran; 3 National Research Center for Genetic Engineering and Biotechnology, Tehran 14155-6343, Iran; 4 Department of Biology, Faculty of Sciences, University of Isfahan, Isfahan 81746-73441, Iran

## Abstract

**Background:**

Salinity is one of the most widespread agricultural problems in arid and semi-arid regions that makes fields unproductive, and soil salinization is a serious problem in the entire world. To determine the effects of salt stress on soybean seedlings, a proteomic technique was used.

**Results:**

Soybean plants were exposed to 0, 20, 40, or 80 mM NaCl for one week. The effect of treatment at 20 mM NaCl on plant growth was not severe, at 80 mM NaCl was lethal, and at 40 mM NaCl was significant but not lethal. Based on these results, proteins were extracted from the leaves, hypocotyls and roots of soybean treated with 40 mM NaCl. Nineteen, 22 and 14 proteins out of 340, 330 and 235 proteins in the leaves, hypocotyls and roots, respectively, were up- and down-regulated by NaCl treatment. In leaves, hypocotyls and roots, metabolism related proteins were mainly down-regulated with NaCl treatment. Glyceraldehyde-3-phosphate dehydrogenase was down-regulated in the leaf/hypocotyls, and fructokinase 2 was down-regulated in the hypocotyls/root with NaCl treatment. Stem 31 kDa glycoprotein precursor was up-regulated in all three organs with NaCl treatment. Glyceraldehyde-3-phosphate dehydrogenase was specifically down-regulated at the RNA and protein levels by salt stress.

**Conclusion:**

These results suggest that metabolism related proteins play a role in each organ in the adaptation to saline conditions.

## Background

Soybean is an important dicot crop due to the high content of oil and protein in its seeds [1]. However, soybean is subject to abiotic stresses that reduce its yield like many crops. Salinity is one of the most widespread agricultural problems in arid and semi-arid regions that makes fields unproductive, and soil salinization is a serious problem in the entire world [2]. Salt stress severely inhibits plant growth for two reasons: first by an osmotic or water-deficit effect of salinity and second by a salt-specific or ion-excess effect of NaCl. However, plants suffer from composite stresses caused by salinity, including water deficit and ion imbalance [3]. Adaptation to salt stress requires alterations in gene expression and subsequently the protein profile of the plant and is very complicated at the whole plant and cellular levels [4,5].

Some salt-inducible genes have been investigated in soybean. A homologue of oxysterol-binding protein was involved in the salt-stress response and cotyledon senescence of soybean [6]. An acidic isoform of pathogenesis-related protein group 5 (PR-5) that is responsive to high salt stress and dehydration was located in the extracellular space of soybean roots [7]. A leucine-zipper-like protein was induced under salt stress and acted in mature organs of soybean shoots to counteract water-potential changes [8]. An acid phosphatase was related to the adaptation of soybean to salt stress, and was involved in reactive oxygen species formation or scavenging or in stress-responsive signal transduction pathways [9]. The overexpression of a dehydration responsive element binding protein homologous gene (*GmDREB2*) in soybean caused the accumulation of a higher level of free proline compared to wild-type plants under salt stress; this gene also was an important transcriptional activator and was useful in improving plant tolerance to salt stress [10]. These salt stress-induced genes may lead to up-regulation or down-regulation of salt stress related proteins [3].

Abbasi and Komatsu [11] studied salt responsive proteins in rice using a proteomic technique, which indicated that an oxygen evolving enhancer protein expressed in the leaf sheath and leaf blade of rice showed a coordinated response to salt stress. Proteomics is a powerful molecular tool for describing the complete proteome at the organelle, cell, organ, or tissue level and for comparing how the proteome is affected by different physiological conditions. Two-dimensional polyacrylamide gel electrophoresis (2-DE) is one of the most sensitive and powerful techniques for resolving hundreds of proteins [12]. 2-DE has been applied to different abiotic treatments for soybean including treatment with salt [13], flooding [14] and exposure to ultraviolet radiation [15]. Proteomic responses of citrus [16], *Salicornia europaea *[17], *Bruguiera gymnorhiza *[18], *Suaeda Aegyptiaca *[19] and rice [20] to salt stress have been reported.

Only one proteomics study has been reported on salt stress in soybean. Aghaei et al. [11] identified salt responsive proteins using a proteomic technique concluding that especially late embryogenesis-abundant protein was involved in the process of adaptation to salt stress at the early seedling stage. Aghaei et al. [11] used 3-day-old seedlings and 100 mM NaCl and proteins were extracted from combined roots and hypocotyls. Soybean seedlings suffer from NaCl and it is very important to improve salt tolerance on seedlings for transition from vegetative to reproductive stage on the farm [1,21]. In this study, proteome analysis was performed on leaf, hypocotyl and root of 7-day-old seedlings to determine the importance of salt-responsive proteins in vegetative stage. Proteins from these organs were separated by 2-DE, and the major differentially expressed proteins were identified by protein sequencer and mass spectrometry (MS).

## Results

### The effect of 40 mM NaCl on soybean seedlings is significant but not lethal

**Figure 1:**
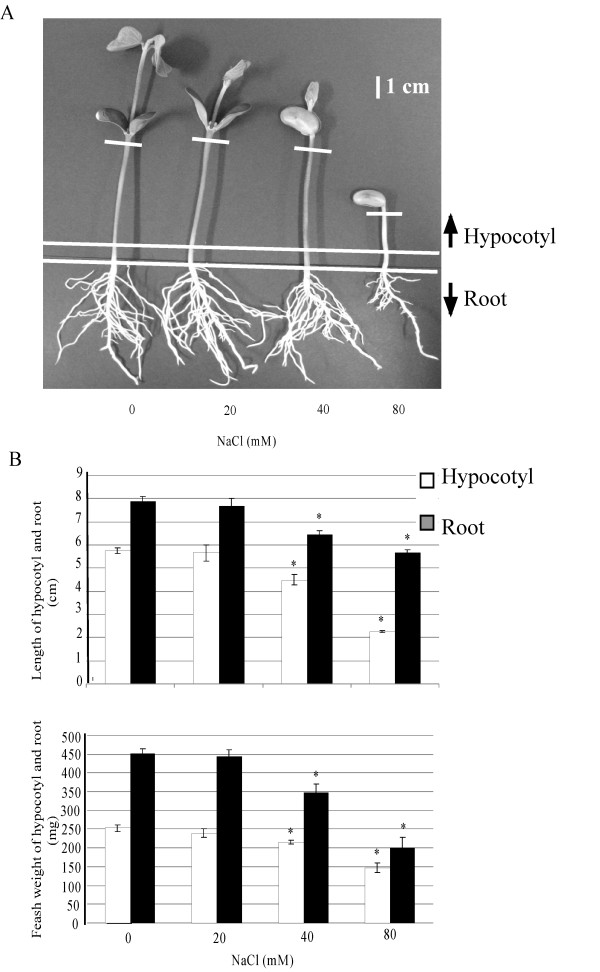
**The effect of NaCl on soybean growth**. Seeds were sown and treated with or without 20, 40, or 80 mM NaCl. The photograph was taken 7 days after the start of treatment (A) and the length and fresh weight of the hypocotyls (white columns) and roots (black columns) were measured (B). For each experiment, 5 soybean plants were used. Values are the mean ± SE from 3 independent experiments. Asterisks indicate significant based on t-test (*P < 0.05) differences between control and NaCl treatments.

To evaluate the effects of salt stress, soybean seeds were sown and treated with 0, 20, 40 or 80 mM NaCl. Morphological changes were examined (Figure 1A) and the length and fresh weight of the hypocotyl and root of 7-day-old seedlings were measured (Figure 1B). After 7 days, first and primary leaves had opened in control plants, whereas they remained closed in treatment with 20 and 40 mM NaCl. There was no first leaf in plants treated with 80 mM NaCl (Figure 1A). The length of the hypocotyl and root was shorter in NaCl treatment. The length of hypocotyl and root of plant treated with 80 mM NaCl was 60% and 28% shorter, respectively, than control plants. The shorter length of hypocotyl and root was significant for 40 mM NaCl compared to the control plants. The fresh weight of hypocotyls and roots from plants treated with 80 mM NaCl was 42% and 56% of the control plants, respectively. The fresh weight of hypocotyls and roots was significantly lower at 40 mM NaCl compared to the control plants. The effect of treatment at 20 mM NaCl on plant growth was not severe even after 7 days, the effect of treatment at 40 mM NaCl was significant but not lethal, and the effect of treatment at 80 mM NaCl was lethal (Figure 1B).

### Na and K contents in the leaf, hypocotyl and root of soybeans treated with NaCl

To investigate the effect of salt stress on the K and Na contents in soybean, the concentrations of these ions were measured in root, hypocotyls and leaf of soybean at 0, 20, 40 and 80 mM NaCl after 2 weeks (see Additional file 1). At 40 mM NaCl, the Na content of the leaf, hypocotyle and root increased, but K content did not changed under NaCl treatment (see Additional file 1).

### In the leaf, hypocotyl and root, 19, 22 and 14 proteins are changed, respectively, in response to 40 mM NaCl treatment

**Figure 2:**
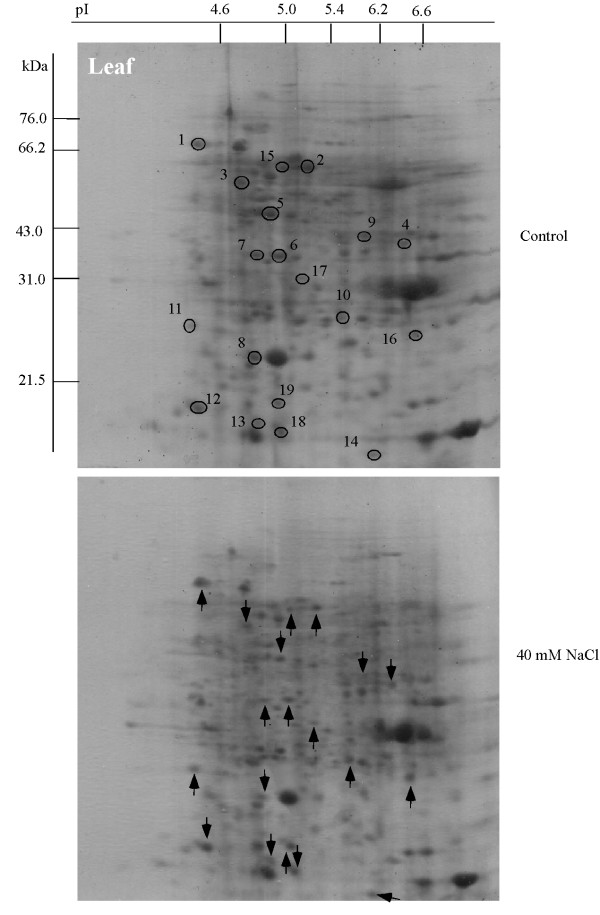
**Protein expression patterns in the leaf of soybeans treated with NaCl**. Seeds were sown and treated with 40 mM NaCl. After 7 days, proteins were extracted from the leaf separated by 2-DE, and visualized by CBB staining. The pI and Mr of each protein were determined using 2-DE markers (Bio-Rad). Arrows indicate protein changes due to NaCl treatment, and circles mark the position of the same proteins from the control. Upward arrows indicate up-regulation and downward arrows indicate down-regulation.

**Figure 3:**
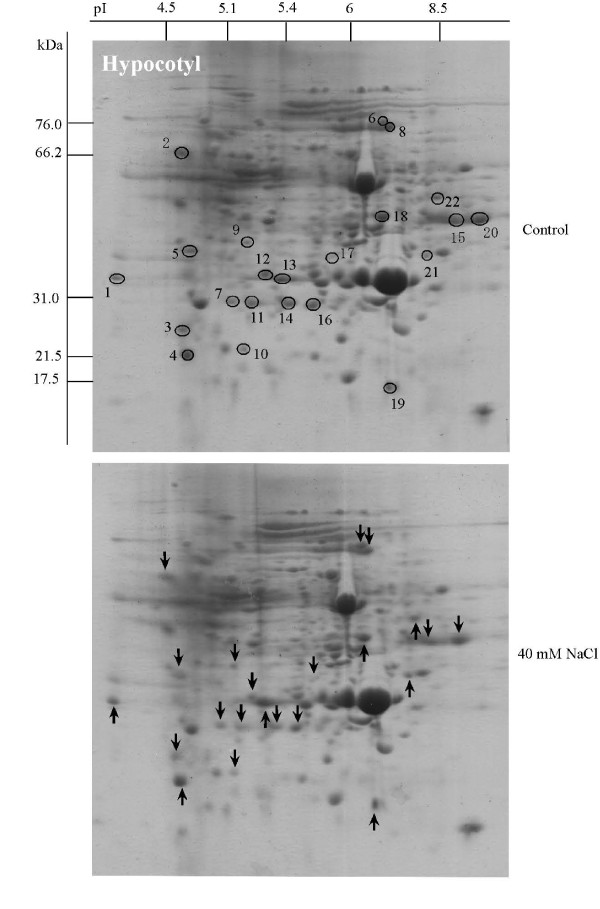
**Protein expression patterns in the hypocotyl of soybeans treated with NaCl**. Seeds were sown and treated with 40 mM NaCl. After 7 days, proteins were extracted from the hypocotyl separated by 2-DE, and visualized by CBB staining. The pI and Mr of each protein were determined using 2-DE markers (Bio-Rad). Arrows indicate protein changes due to NaCl treatment, and circles mark the position of the same proteins from the control. Upward arrows indicate up-regulation and downward arrows indicate down-regulation.

**Figure 4:**
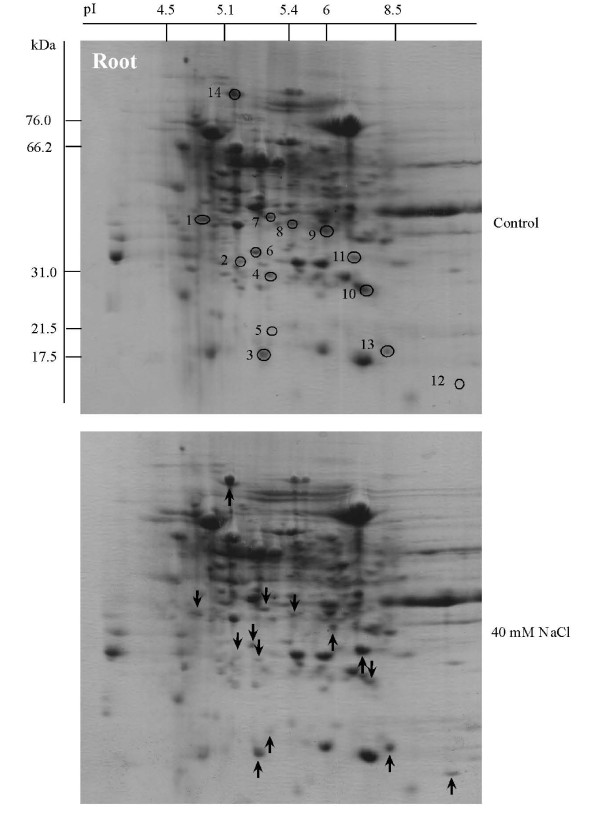
**Protein expression patterns in the root of soybeans treated with NaCl**. Seeds were sown and treated with 40 mM NaCl. After 7 days, proteins were extracted from the root separated by 2-DE, and visualized by CBB staining. The pI and Mr of each protein were determined using 2-DE markers (Bio-Rad). Arrows indicate protein changes due to NaCl treatment, and circles mark the position of the same proteins from the control. Upward arrows indicate up-regulation and downward arrows indicate down-regulation.

Soybean seeds were sown and treated with 40 mM NaCl, then proteins were extracted from the leaf, hypocotyl and root, separated by 2-DE, and stained with CBB to evaluate their expression level. Using PDQuest software analysis, 340, 330 and 235 protein spots were reproducibly detected on the 2-DE gels of leaf, hypocotyl and root, respectively (Figures 2, 3 and 4, upper panels), with 11, 12 and 7 proteins visibly up-regulated and 8, 10 and 7 proteins down-regulated by 40 mM NaCl treatment (Figures 2, 3 and 4, lower panels, see Additional files 2, 3 and 4). Aghaei et al. [13] reported that 321 protein spots were detectable from 2-DE gels for 3-day-old soybean seedlings. Among these spots, 7 proteins were up- or down-regulated under salt stress.

### Photosynthesis related proteins are down-regulated in soybean leaves by NaCl treatment

**Figure 5:**
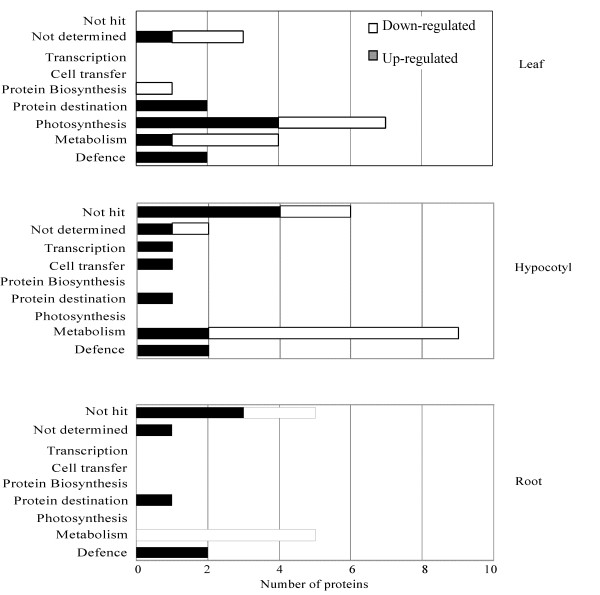
**Functional distribution of identified proteins expressed in leaf, hypocotyl and root of soybean seedlings after NaCl treatment**. After identification of proteins up- (black columns) and down- (white columns) regulated with NaCl treatment, the proteins were classified into 9 functional groups, which are: metabolism, photosynthesis, defense, cell transfer, protein destination and storage, protein synthesis, transcription, not hit and not determined.

In the leaf, 11 proteins were identified by N-terminal sequencing and 5 proteins were identified by MALDI-TOF MS (see Additional files 2 and 5). These proteins were calreticulin (L01), ATP synthase CF1 beta subunit (L02), glyceraldehyde-3-phosphate dehydrogenase (L04), RuBisCO activase (L05), oxygen-evolving enhancer protein 1 (L06 and 07), fructose-bisphosphate aldolase (L09), 20-kDa chaperonin (L10), protease inhibitor precursor (L11), 50S ribosomal protein L12-3 (L12), stem 31 kDa glycoprotein precursor (L19) and RuBisCO small (L14) and large subunits (L03, L13, L17 and L18). In the leaf, proteins involved in metabolism (1 protein), defense (2 proteins), protein destination/storage (2 proteins) and photosynthesis (4 proteins) were up-regulated, whereas proteins involved in photosynthesis (3 proteins) and metabolism (3 proteins) were down-regulated by NaCl treatment (Figure 5).

### Metabolic related proteins are down-regulated in soybean hypocotyls by NaCl treatment

In hypocotyls, 6 proteins were identified by N-terminal sequencing and 14 proteins were identified by MALDI-TOF MS (see Additional files 3 and 6). These proteins were kinesin motor protein (H01), NADH dehydrogenase 1 beta subcomplex subunit 8 (H03), trypsin inhibitor B (H04), methionine synthase (H06), transketolase (H08), putative fructokinase 2 (H09), DNA-directed RNA polymerase gamma chain (H10), acetoacetyl-CoA reductase (H11), stem 31 kDa glycoprotein precursor (H13), triosephosphate isomerase (H14), glyceraldehyde-3-phosphate dehydrogenase (H15), acid phosphatase (H16), alcohol dehydrogenase Adh-1 (H18) and annexin (H21). In hypocotyls, proteins involved in defense (2 proteins) and metabolism (2 proteins) were up-regulated, whereas proteins involved in metabolism (7 proteins) were down-regulated by NaCl treatment (Figure 5).

### Metabolic related proteins are down-regulated in soybean roots by NaCl treatment

In the root, 13 proteins were identified by MALDI-TOF MS (see Additional files 4 and 7). These proteins were putative fructokinase 2 (R01), dienelactone hydrolase family protein (R02), caffeoyl-CoA-O-methyltransferase (R06), NADPH:isoflavone reductase (R07), putative cinnamyl alcohol dehydrogenase (R09), putative quinone oxidoreductase (R10), stem 31 kDa glycoprotein precursor (R11) and ripening related protein (R13). In the root, proteins involved in defense (2 proteins) were up-regulated, whereas proteins involved in plant metabolism (5 proteins) were down-regulated by NaCl treatment (Figure 5).

### Proteins in common among leaves, hypocotyls and roots show organ and developmental stage specificity at the mRNA level

**Figure 6:**
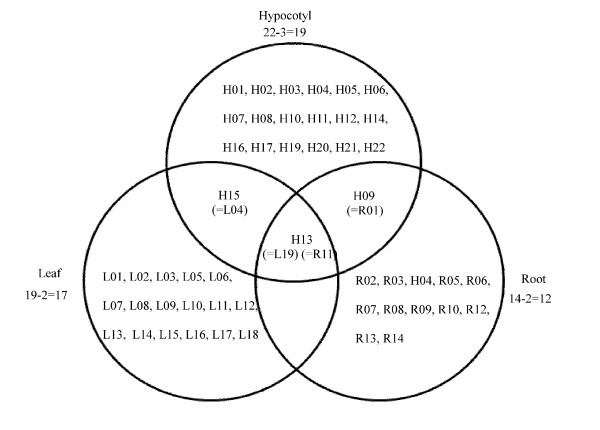
**Venn diagram analysis showing up- or down-regulated proteins that overlapped between the leaf, hypocotyl and root of soybean seedlings**. Effects of salt stress on these three parts of soybean seedlings were analyzed. Numbers correspond to the protein spots in the 2-DE patterns from the leaf, hypocotyl and root.

A total of 19, 22 and 14 proteins were changed by 40 mM NaCl treatment in the leaf, hypocotyl and root, respectively. Three of these proteins were involved in at least two of these organs with salt stress. These proteins were glyceraldehyde-3-phosphate dehydrogenase (H15, L04), stem 31 kDa glycoprotein precursor (H13, L04, R11) and putative fructokinase 2 (H09, R01) (Figure 6). The remaining proteins were organ specific, suggesting that salt stress affects organ specific proteins by up- or down-regulation. These results indicate that three proteins are mostly affected by NaCl treatment. To identify whether these genes coding proteins are expressed with organ specificity, RNA expression levels were examined. Total RNA was extracted from 3- and 5-day-old hypocotyls and from 7-day-old leaves, hypocotyls and roots of soybean seedlings and quantitative real-time PCR was then performed.

**Figure 7:**
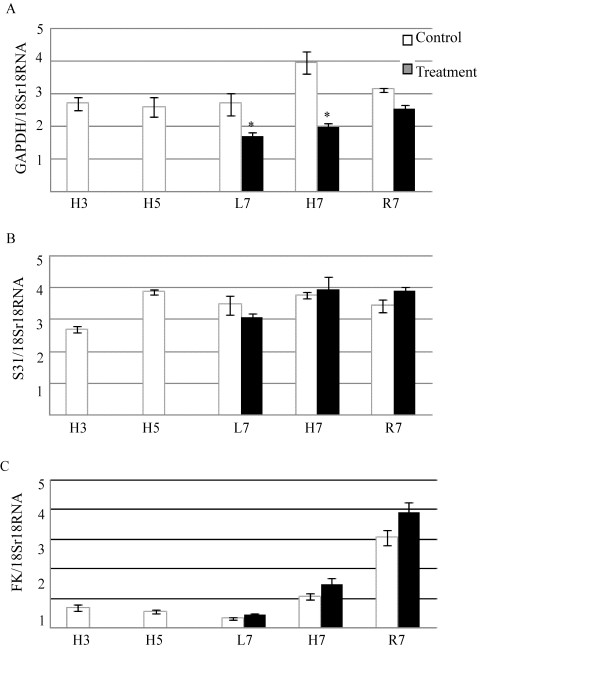
**Relative amount of mRNA for salt responsive genes in leaf, hypocotyl and root of soybean seedlings**. Total RNA was extracted from 3- and 5-day-old hypocotyls and from 7-day-old leaves, hypocotyls and roots of soybean seedlings after 40 mM NaCl treatment. cDNA was synthesized by reverse transcription, and quantitative real-time PCR was performed using the cDNA products. An 18S rRNA cDNA clone was used as a template to produce a standard curve and the relative amount of mRNA was calculated by the ΔCt method. Values are the mean ± SE from 3 independent experiments and asterisks indicate significant (P < 0.05) differences between control (white columns) and NaCl treatments (black columns). *GAPDH*, glyceraldehyde-3-phosphate dehydrogenase; *S31*, stem 31 kDa glycoprotein precursor; *FK*, putative fructokinase 2; L, leaf; H, hypocotyl; R, root; H3, 3-day-old hypocotyl and H5, 5-day-old hypocotyl.

The relative amount of glyceraldehyde-3-phosphate dehydrogenase mRNA was decreased in the leaves, hypocotyls and roots relative to controls by NaCl treatment. The decrease was significant for hypocotyls and roots (Figure 7A). The expression of this protein was down-regulated in leaves and hypocotyls (see Additional files 5 and 6). These results indicate that the down-regulation of glyceraldehyde-3-phosphate dehydrogenase is caused at both the mRNA and protein level by NaCl treatment. On the other hand, fructokinase 2 was differentially expressed in the hypocotyl and root under salt stress (Figure 6). The relative amount of putative fructokinase 2 mRNA was increased in the leaf, hypocotyl and root, but not significantly (Figure 7C). At the protein level, this protein was down-regulated in the hypocotyl and root (see Additional files 6 and 7). Fructokinase catalyzes the phosphorylation of fructose to fructose-6-phosphate. Fructose-6-phosphate is a major substrate for many sugar metabolic pathways including glycolysis, starch biosynthesis, and the oxidative pentose pathway [22]. Under salt stress, by inhibiting photosynthesis, the amount of fructose-6-phosphate decreases, because of the decrease in photosynthetic sucrose. Fructokinase was down-regulated in this study, presumably as a result of negative feedback caused by a decrease in the amount of fructose-6-phosphate.

The relative amount of stem 31 kDa glycoprotein precursor mRNA was increased in the hypocotyl and root but decreased in the leaf by NaCl treatment. The changes for stem 31 kDa glycoprotein precursor mRNA were not significant among the three organs (Figure 7B). The expression of this protein was up-regulated in all three organs (see Additional files 5, 6 and 7). Aghaei et al. [13] reported that the expression of stem 31 kDa glycoprotein precursor was down-regulated in hypocotyls and roots of 3-day-old soybean seedlings by NaCl treatment. In this study, the amount of stem 31 kDa glycoprotein precursor mRNA was increased in the hypocotyl of 3-day-old to 5-day-old soybean seedlings (Figure 7B). Based on this study, after 3 days there is a developmental increase in the amount of stem 31 kDa glycoprotein precursor mRNA. Stem 31 kDa glycoprotein precursor may function as a somatic storage protein during early seedling development and it accumulates mainly in the stems of developing soybean seedlings [23,24]. Its differential change in different organs of soybean seedlings under salt stress indicates its rate of consumption from storage and *de novo *biosynthesis during development. At the protein level, this protein was up-regulated in all three organs examined, which indicates the decrease in consumption of this storage protein caused by the decrease in plant growth under salt stress.

## Discussion

Growth in length and fresh weight of the hypocotyls and roots was completely abolished by 80 mM NaCl treatment. It was reported by Aghaei et al. [13] that growth was inhibited by 100 mM NaCl treatment of 3-day-old soybean seedlings. Based on the present study and the report of Aghaei et al. [13], it is improved that soybean is a relatively NaCl sensitive crop and that these deleterious effects of NaCl are mainly due to up- or down-regulation of genes and their corresponding proteins following NaCl treatment. In this study, Na content was increased under salt stress, but K content did not decrease (see Additional file 1). This result suggests that Na toxicity leads to damaging effects of NaCl in soybean. In order to investigate the effects of NaCl on 7-day-old soybean seedlings, the leaf, hypocotyl and root of 40 mM NaCl-treated seedlings were used for proteome analysis.

Proteins changed in soybean leaves by salt stress show that photosynthesis related proteins are mainly down-regulated and suggest that NaCl affects photosynthesis and leads to energy reduction inside the plant and consequent reduction in plant growth. Calreticulin is an important calcium-binding protein with chaperone functions and plays a pivotal role in regulating calcium homeostasis and protein folding in the endoplasmic reticulum of plants [25,26]. Calreticulin was down-regulated in rice under osmotic stress [27]. This indicates the main role of calcium as a main secondary messenger for soybean seedlings under salt stress. The principal role of RuBisCO activase is to release inhibitory sugar phosphates such as ribulose-1,5-biphosphate from the active sites of RuBisCO so that CO_2 _can activate the enzyme by carbamylation [28]. RuBisCO activase also has another role as a chaperone during stress [29]. RuBisCO activase was down-regulated by NaCl and this led to the main inhibitory effect of NaCl on soybean photosynthesis [5]. The 20-kDa chaperonin functions only as a co-chaperone, along with cpn60, and in certain cases is essential for the discharge of biologically active proteins from cpn60 [30]. Chaperones act to repair the potential damage caused by misfolding of proteins. Most newly synthesized proteins can fold in the absence of chaperones, but a minority strictly requires them [31]. The 20-kDa chaperonin was up-regulated in this study, suggesting that protection of proteins by the chaperone in soybean is very important to prevent misfolding of proteins under salt stress. The large 50S ribosomal subunit catalyses the peptidyl-transfer reaction of messenger RNA-directed protein biosynthesis [32]. Down-regulation of the 50S ribosomal protein indicates the inhibitory effect of NaCl on soybean protein biosynthesis and presumably leads to the consequent reduction in plant growth (Figure 1).

Proteins changed in soybean hypocotyls by salt stress show that metabolic related proteins in the hypocotyl of soybean seedlings are mainly affected by down-regulation under salt stress. Kinesin proteins are a large family of plus- or minus-end-directed microtubule motors important in intracellular transport, mitosis, meiosis, and development [33]. Control and maintenance of the cell cycle and cell integrity are critically important for cell-to-cell communication and signaling, especially under stress [34]. Kinesin motor protein was up-regulated, suggesting that it maintains the cell cycle and cell integrity under salt stress. NADH dehydrogenase 1 beta subcomplex subunit 8 is an accessory subunit of the mitochondrial membrane respiratory chain NADH dehydrogenase, which is not involved in catalysis. This protein functions in the transfer of electrons from NADH to the respiratory chain. The immediate electron acceptor for the enzyme is ubiquinone [35]. In this study, NADH dehydrogenase 1 beta subcomplex subunit 8 was down-regulated, suggesting that this led to a decrease in the ATP pool of the soybean seedling cells and a subsequent decrease in plant growth under salt stress. Trypsin inhibitor is capable of reducing dehydroascorbate when in the reduced form but acquires trypsin-inhibiting activity in the oxidized state [36]. Proteolysis would be accelerated under stress and in response to stress conditions, accumulation of trypsin inhibitors will occur [37]. Trypsin inhibitor was up-regulated and this indicates its main role in prevention of protein proteolysis and in H_2_O_2 _detoxification in soybean seedlings under salt stress. The role of alcohol dehydrogenase in tolerance of crops to flooding stress has already been identified [38]. Under stress, plant respiration shifts from aerobic to anaerobic conditions to produce ATP via the fermentative pathway. Alcohol dehydrogenase was up-regulated in the hypocotyl in this study, indicating the main role of this enzyme produce ATP and consume glycolytic products under salt stress. The role of annexin is important for integrity of the cell membrane and cell signaling, and especially under stress conditions, this protein family can help the plant to tolerate the stress and maintain the integrity of the cells [39,40]. Annexin was up-regulated in this study, which indicates the main role of this protein in tolerance of salt stress in the soybean seedling hypocotyl.

Proteins changed in soybean roots by salt stress show that metabolic related proteins in the roots of soybean seedling are mainly affected by down-regulation under salt stress. Dienelactone hydrolase hydrolyzes the conversion of dienelactone to maleylacetate, which both intermediates for aerobic degradation of haloaromatic compounds [41,42]. A dienelactone hydrolase family protein was down-regulated in this study and this could cause a decrease in degradation of these secondary metabolites under salt stress. Caffeoyl-CoA-O-methyltransferase can catalyze the conversion of caffeoyl-CoA to sinapoyl-CoA [43]. These products are the intermediates for lignification of the cell wall and down-regulation of this enzyme may indicate a reduction in cell wall lignification and a consequent decrease in soybean seedling growth under salt stress (Figure 1). Isoflavone reductase catalyzes the NADPH-dependent reduction of 2'-hydroxyformononetin to vestitone, which is the penultimate step in the synthesis of medicarpin in general flavonoid biosynthesis [44]. Down-regulation of this enzyme indicates that flavonoid compounds do not have a main role in tolerance to salt stress in soybean seedlings. Quinone is the product of polyphenol oxidase and is converted to melanin after polymerization [45]. Quinone oxidoreductase was down-regulated and this could be due to a reduction in quinone production or in the amount of phenol compounds in soybean seedling roots under salt stress. Ripening related proteins are involved in plant maturation and ripening [46]. A relationship between the stress hormone ethylene and plant ripening has already been reported [47]. Ethylene induces ripening related proteins and leads the plant to mature and ripen fruit sooner than usual under stress. Up-regulation of ripening related protein could be due to an increase in ethylene concentration of soybean seedlings under salt stress.

Proteome responses of soybean as a salt-sensitive crop to salt stress are different compared to other plants that are mentioned in present study. In soybean, metabolism related proteins were mainly down-regulated with NaCl treatment, but for *Salicornia europaea*, energy production and ion homeostasis associated proteins played important roles for this plant salt tolerance ability [17] or overlapping of hydrogen peroxide and nitric oxide caused the acclimation of citrus plants to salt stress [16]. For *Suaeda aegyptiaca *as a halophytes plant, proteins associated with salt tolerance such as proteins involved in glycinebetain synthesis and oxidative stress tolerance had main roles to tolerate salt stress [19]. Caffeoyl-CoA-O-methyltransferase was up-regulated by salt stress at rice [20], but for present study it was down-regulated at roots (Table 1C). Lignification changes are important in protecting plants from abiotic stress. This indicates different strategies and subsequently different salt tolerant potential between soybean and other plants.

Based on this study, stem 31 kDa glycoprotein precursor shows developmental specificity and putative fructokinase 2 shows organ specificity in response to NaCl treatment. Glyceraldehyde-3-phosphate dehydrogenase was down-regulated at both the mRNA and protein levels in response to NaCl treatment, suggesting that it plays a role in salt stress and can be used as a target gene in soybean seedlings. Jeong et al. [48] improved salt tolerance of potato by transfer of the glyceraldehyde-3 phosphate dehydrogenase gene. This gene has a main role in tolerance to salt stress and there are several reports related to its relationship to improved salt tolerance in plants [49]. Under salt stress, which inhibits photosynthesis, the substrates for glycolysis decrease and there is a resulting decrease in the rate of the glycolytic reactions. At the step catalyzed by glyceraldehyde-3-phosphate dehydrogenase in glycolysis, one NADH is produced from NAD^+^. It is indicated that the ATP production will be reduced by down-regulation of glyceraldehydes-3-phosphate dehydrogenase and consequently there will be a decrease in plant growth under salt stress.

## Conclusions

The growth of 7-day-old soybean seedlings was affected and their protein expression was changed in the leaf, hypocotyl and root by salt stress. Glyceraldehyde-3-phosphate dehydrogenase was down-regulated at both the protein and mRNA levels in leaves and hypocotyls of soybean seedling by salt stress (Figure 7, see Additional files 5, 6 and 7). ATP production will be reduced by down-regulation of glyceraldehydes-3-phosphate dehydrogenase and consequently there will be a decrease in plant growth under salt stress. Based on previous and present study, this gene can be one of the target genes to improve salt tolerance of soybean in the future.

## Methods

### Plant materials and treatment

Seeds of soybean (*Glycin max *[L.] Merr. cv. Enrei) were sown on the sand and then treated with 0, 20, 40 or 80 mM NaCl. They were grown in a growth chamber under white fluorescent light (600 μmol m^-2 ^s^-1^; 16 h light/8 h dark) at 25°C and 70% relative humidity. Length and fresh weight of hypocotyls and roots of healthy seedlings were measured 7 days after sowing. Physiologically independent experiments were carried out 3 times, and 5 soybean plants were used for each experiment.

### Analysis of Na and K contents

Freeze-dried hypocotyls, roots and leaves were used for analysis of Na and K contents. The samples were powdered by grinding with pestl and mortar. A portion of the powered samples were digested with concentrated HNO_3 _at 110°C for 2 h. Na and K contents in the digested samples were determined by atomic absorption.

### Protein extraction

A portion (200 mg and 500 mg, respectively) of hypocotyls and roots from soybean was homogenized in phosphate saline buffer (pH 7.6) containing 65 mM K_2_HPO_4_, 2.6 mM KH_2_PO_4_, 400 mM NaCl and 3 mM NaN_3 _at 4°C using a glass mortar and pestle on ice. The homogenate was centrifuged at 15,000 × g for 10 min, and then cold trichloroacetic acid was added to the supernatant to a final concentration of 10%. The solution was kept on ice for 30 min and then centrifuged for 10 min at 15,000 × g at 4°C. The resultant precipitate was homogenized with 100 μL of lysis buffer containing 8.5 M urea, 2.5 M thiourea, 5% CHAPS, 100 mM dithiothreitol and 0.5% Bio-Lyte (pH 3.0-10.0 and pH 5.0-8.0) in a glass mortar and pestle on ice. A portion (200 mg) of leaf was directly homogenized in lysis buffer containing 7 M urea, 0.2 M thiourea, 0.2 mM TBP, 0.4% CHAPS, 0.2% Bio-Lyte (pH 3.0-10.0) and 5% polyvinylpyrrolidone 40. All homogenates were centrifuged twice at 15,000 × g at room temperature for 10 min. The supernatant was subjected to electrophoresis.

### Two-dimensional polyacrylamide gel electrophoresis

Proteins were separated by 2-DE in the first dimension by isoelectric focusing tube gel (11 cm) and in the second dimension by SDS-PAGE [12]. For the first dimension, the isoelectric focusing gel consisted of 8 M urea, 3.5% polyacrylamide, 2% Nonidet P-40, 2% Bio-Lyte (pH 3.0-10.0 and pH 5.0-8.0), ammonium peroxodisulfate and tetramethylethylenediamine. Electrophoresis was carried out at 200 V for 30 min, followed by 400 V for 16 h and 600 V for 1 h. After separation, SDS-PAGE as the second dimension was performed using a 15% polyacrylamide gel (160 × 140 × 1 mm) with a 5% stacking gel. The gels were stained with Coomassie brilliant blue (CBB) and image analysis was performed. 2-DE analysis was done 5 times for independent samples. For each sample 2-DE gels were casted at least 2 times.

### Image acquisition and data analysis

Three gels out of 5 gels were used for image acquisition and data analysis. Spot detection, spot measurement, background subtraction and spot matching were performed after CBB staining of the gels using PDQuest software (version 7.1, Bio-Rad, Hercules, CA, USA). Following automatic spot detection, gel images were carefully edited. Before spot matching, one of the gel images was selected as a reference gel. The amount of a protein spot was expressed as the volume of that spot, which was defined as the sum of the intensities of all the pixels that make up the spot. In order to correct the variability due to CBB staining and to reflect the quantitative variation in intensity of protein spots, the spot volumes were normalized as a percentage of the total volume in all spots in the gel. The resulting data from image analysis were transferred to PDQuest software for querying protein spots that showed quantitative and qualitative variations. The pI and Mr of each protein were determined using 2-DE markers (Bio-Rad).

### N-terminal amino acid sequence analysis and homology searches

Following separation by 2-DE, proteins were electroblotted onto a polyvinylidine difluoride (PVDF) membrane (Pall, Port Washington, NY, USA) using a semidry transfer blotter (Nippon Eido, Tokyo, Japan), and detected by CBB staining. The stained protein spots were excised from the PVDF membrane and applied to the upper glass block of the reaction chamber in a gas-phase protein sequencer (Procise cLC, Applied Biosystems, Foster City, CA, USA). Edman degradation was performed according to the standard program supplied by the manufacturer. The amino acid sequences obtained were compared with those of known proteins in the Swiss-Prot, PIR, GenPept and PDB databases with the Web-accessible search program FASTA http://www.dna.affrc.go.jp.

### Analysis using matrix-assisted laser desorption ionization time-of-flight mass spectrometry

Proteins were excised from 2-DE gels stained by CBB and then alkylation and protein digestion with trypsin were done using a DigestPro96 robotic system (Intavis AG, Koeln, Germany). The generated peptides were purified using NuTip C-18 columns (Glygen Corp., Columbia, MD, USA). The purified peptides were added to an α-cyano-4-hydroxycinamic acid matrix and dried onto a plate for analysis using a matrix-assisted laser desorption ionization time-of-flight (MALDI-TOF) MS (Voyager-DE RP, Applied Biosystems). Calibration was external and data were collected in the reflector mode. Data were searched on the Internet using an in-house licensed Mascot search engine (version 2.2.18, Matrix Science, Ltd., London, UK) against all entries in the soybean genome database (version 4, 62,199 sequences), which was especially constructed for this research based on preliminary soybean genome sequences from the Department of Energy (DOE) Joint Genome Institute and the Soybean Genome Sequencing Consortium. Genome sequences were downloaded from the DOE database (http://www.phytozome.net, release data 24 January, 2008) and then converted into FASTA format. Carbamidomethylation of cysteines was set as a fixed modification and oxidation of methionines was set as a variable modification. Trypsin was specified as the proteolytic enzyme and one missed cleavage was allowed. Identified proteins with a peptide mass fingerprint were denoted as having an unambiguous identification by the following criteria: (1) the deviation between the experimental and theoretical peptide masses needed to be less than 50 ppm; (2) at least five different predicted peptide masses were needed to match the observed masses for an identification to be considered valid; (3) the matching peptides needed to cover at least 10% of the known protein sequence; and (4) protein scores needed to have > 60 identity for soybean DOE database and > 81 identity for NCBI database (p < 0.05).

### RNA extraction and quantitative RT-PCR from the leaf, hypocotyl and root of soybean seedlings

Total RNA was extracted from 100 mg each of leaves, hypocotyls and roots of soybean seedlings using an RNeasy plant mini kit with DNAse (Qiagen, Valencia, CA, USA) treatment. A portion (800 ng) of total RNA from each sample was reverse-transcribed to cDNA in a 20 μL reaction volume using iScript cDNA synthesis kit (Bio-Rad) according to the manufacturer's protocol. Real-time quantitative PCR was performed using the cDNA product corresponding to 20 ng of total RNA in a 20 μL reaction volume using the iQ SYBR Green supermix (Bio-Rad) on a MyiQ single-color real-time PCR detection system (Bio-Rad). The PCR conditions were as follows: 95°C for 3 min, then 45 cycles of 95°C for 30 sec, 58°C for 30 sec and 72°C for 30 sec. To normalize gene expression, 18S rRNA (X02623) was used as an internal control. A dilution series of 18S rRNA cDNA clone and the cDNA products were used as templates to confirm amplification effieciency. The relative amount of mRNA was calculated by the ΔCt calculation [50]. The calculation was performed by following calculation formula: 2^(threshold cycle of 18S rRNA-threshold cycle of target gene)^·10^8 ^. The primers were designed using the Primer3 web interface http://frodo.wi.mit.edu/[51]. Primer sets for the genes examined had the following sequences: for the stem 31 kDa glycoprotein precursor, the forward sequence was ACCTTCACCTCTCTCAACAATC and the reverse sequence was AAAGCCGCGTAAGAGACAAC; for glyceraldehyde-3-phosphate dehydrogenase, the forward sequence was GAGGGGGATCGAGTTTTTG and the reverse sequence was CACGAATCCATGATTCAAAGC; for the putative fructokinase 2, the forward sequence was AGAGGAGGCTGCACTGAAAC and the reverse sequence was GCGTTATTTGGCAAAACCTG and for 18S rRNA, the forward sequence was TGATTAACAGGGACAGTCGG and the reverse sequence was ACGGTATCTGATCGTCTTCG.

## Abbreviations

2-DE: two-dimensional polyacrylamide gel electrophoresis; CBB: coomassie brilliant blue; PVDF: polyvinylidene difluoride; RuBisCO: ribulose-1,5-bisphosphate carboxylase/oxygenase; MALDI-TOF MS: matrix-assisted laser desorption ionization time-of-flight mass spectrometry.

## Competing interests

The authors declare that they have no competing interests.

## Authors' contributions

HS carried out sample preparation, 2DE and structure analysis for root and hypocotyls. RR carried out sample preparation, 2DE and structure analysis for leaf. YN carried out quantitative RT-PCR and Na/K content analysis. AAE, ERJ, and NM helped the research. SK conceived, designed, implemented and coordinated the study, and also carried out physiological experiments. All authors read and approved the final manuscript.

## Supplementary Material

Additional file 1 **Na and K contents in the leaf, hypocotyl and root of soybeans treated with NaCl**. Soybeans were spwn on the sand and treated with 0, 20, 40 or 80 mM NaCl. They were grown for 2 weeks, and Na and K contents of leaf, hypocotyls and root were measured. Ten plants in each treatment were used. The experiments were repeated 3 times and the results show the average ± SE. Click here for file

Additional file 2 **Relative levels of protein expression of leaf of soybeans treated with NaCl**. Relative intensities of leaf were obtained using PDQuest software. Values are the mean ± SE. Error bars show SE of 3 spots from 3 independent experiments. The numbers of spots are the same as in Figure 2. White and black columns are the control and NaCl treatments, respectively. Click here for file

Additional file 3 **Relative levels of protein expression of hypocotyl of soybeans treated with NaCl**. Relative intensities of hypocotyl were obtained using PDQuest software. Values are the mean ± SE. Error bars show SE of 3 spots from 3 independent experiments. The numbers of spots are the same as in Figure 3. White and black columns are the control and NaCl treatments, respectively. Click here for file

Additional file 4 **Relative levels of protein expression of root of soybeans treated with NaCl**. Relative intensities of root were obtained using PDQuest software. Values are the mean ± SE. Error bars show SE of 3 spots from 3 independent experiments. The numbers of spots are the same as in Figure 4. White and black columns are the control and NaCl treatments, respectively. Click here for file

Additional file 5 **Salt stress responsive proteins in leaves of soybean seedlings**. a) Spot No, Spot number; b) The sequence shown is the N-terminal amino acid sequence determined by protein sequencing; c) Accession No, Accession number; d) Exp. Mr/pI shows experimental molecular weight and isoelectric point; e) Theor. Mr/pI shows theoretical molecular weight and pH isoelectric; f) PM, Number of matched peptides; g) SC, Sequence coverage by peptide mass fingerprinting using MALDI-TOF MS; h) U & D, up-regulated and down-regulated spots based on significant (*p *< 0.05) differences between control and NaCl treatments; i) CV ± SE, Spot volume of control ± standard error; j) TV ± SE, Spot volume of treatment ± standard error; k) T/C ratio, Treatment spot volume/control spot volume ratio; l) Category shows functional classification; m) ND, Not determined; M, metabolism; P, photosynthesis; D, defence; CT, cell transfer; PD, protein destination and storage; PS, protein synthesis; T, transcription. Click here for file

Additional file 6 **Salt stress responsive proteins in hypocotyls of soybean seedlings**. a) Spot No, Spot number; b) The sequence shown is the N-terminal amino acid sequence determined by protein sequencing; c) Accession No, Accession number; d) Exp. Mr/pI shows experimental molecular weight and isoelectric point; e) Theor. Mr/pI shows theoretical molecular weight and pH isoelectric; f) PM, Number of matched peptides; g) SC, Sequence coverage by peptide mass fingerprinting using MALDI-TOF MS; h) U & D, up-regulated and down-regulated spots based on significant (*p *< 0.05) differences between control and NaCl treatments; i) CV ± SE, Spot volume of control ± standard error; j) TV ± SE, Spot volume of treatment ± standard error; k) T/C ratio, Treatment spot volume/control spot volume ratio; l) Category shows functional classification; m) ND, Not determined; M, metabolism; P, photosynthesis; D, defence; CT, cell transfer; PD, protein destination and storage; PS, protein synthesis; T, transcription. Click here for file

Additional file 7 **Salt stress responsive proteins in roots of soybean seedlings**. a) Spot No, Spot number; b) The sequence shown is the N-terminal amino acid sequence determined by protein sequencing; c) Accession No, Accession number; d) Exp. Mr/pI shows experimental molecular weight and isoelectric point; e) Theor. Mr/pI shows theoretical molecular weight and pH isoelectric; f) PM, Number of matched peptides; g) SC, Sequence coverage by peptide mass fingerprinting using MALDI-TOF MS; h) U & D, up-regulated and down-regulated spots based on significant (*p *< 0.05) differences between control and NaCl treatments; i) CV ± SE, Spot volume of control ± standard error; j) TV ± SE, Spot volume of treatment ± standard error; k) T/C ratio, Treatment spot volume/control spot volume ratio; l) Category shows functional classification; m) ND, Not determined; M, metabolism; P, photosynthesis; D, defence; CT, cell transfer; PD, protein destination and storage; PS, protein synthesis; T, transcription. Click here for file

## References

[B1] Luo Q Yu B Liu Y Differential sensitivity to chloride and sodium ions in seedlings of Glycine max and G. soja under NaCl stress J Plant Physiol 2005 162 1003 1012 16173461 10.1016/j.jplph.2004.11.008

[B2] Sharifi M Ghorbanli M Ebrahimzadeh E Improved growth of salinity-stressed soybean after inoculation with salt pre-treated mycorrhizal fungi J Plant Physiol 2007 164 1144 1151 10.1016/j.jplph.2006.06.016 16919369

[B3] Munns R James RA Lauchli A Approaches to increasing the salt tolerance of wheat and other cereals J Exp Bot 2006 57 1 19 10.1093/jxb/erj100 16510517

[B4] Ashraf M Foolad MR Roles of glycine betaine and proline in improving plant abiotic stress resistance Environ Exp Bot 2007 59 206 221 10.1016/j.envexpbot.2005.12.006

[B5] Parker R Flowers TJ Moore AL Harpham NVJ An accurate and reproducible method for proteome profiling of the effects of salt stress in the rice leaf lamina J Exp Bot 2006 57 1109 1118 10.1093/jxb/erj134 16513811

[B6] Li DY Inoue H Takahashi M Kojima T Shiraiwa M Takahara H Molecular characterization of a novel salt-inducible gene for an OSBP (oxysterol-binding protein)-homologue from soybean Gene 2008 407 12 20 10.1016/j.gene.2007.02.029 17466467

[B7] Onishi M Tachi H Kojima T Shiraiwa M Takahara H Molecular cloning and characterization of a novel salt-inducible gene encoding an acidic isoform of PR-5 protein in soybean (*Glycine max *[L.] Merr.) Plant Physiol Biochem 2006 44 574 580 10.1016/j.plaphy.2006.09.009 17070691

[B8] Aoki A Kanegami A Mihara M Kojima T Shiraiwa M Takahara H Molecular cloning and characterization of a novel soybean gene encoding a leucine-zipper-like protein induced to salt stress Gene 2005 356 135 145 10.1016/j.gene.2005.04.014 15964719

[B9] Liao H Wong FL Phang TH Cheung MY Li WYF Shao G Yan X Lam HM *GmPAP3*, a novel purple acid phosphatase-like gene in soybean induced by NaCl stress but not phosphorus deficiency Gene 2003 318 103 111 10.1016/S0378-1119(03)00764-9 14585503

[B10] Chen M Wang QY Cheng XG Xu ZS Li LC Ye XG Xia LQ Ma YZ *GmDREB2*, a soybean DRE-binding transcription factor, conferred drought and high-salt tolerance in transgenic plants Biochem Biophys Res Commun 2007 353 299 305 10.1016/j.bbrc.2006.12.027 17178106

[B11] Abbasi FM Komatsu S A proteomic approach to analyze salt responsive proteins in rice leaf sheath Proteomics 2004 4 2072 2081 10.1002/pmic.200300741 15221768

[B12] O'Farrell HP High resolution two-dimensional electrophoresis of proteins J Biol Chem 1975 250 4007 4021 236308 PMC2874754

[B13] Aghaei K Ehsanpour AA Shah AH Komatsu S Proteome analysis of soybean hypocotyl and root under salt stress Amino Acids 2009 36 91 98 10.1007/s00726-008-0036-7 18264660

[B14] Shi F Yamamoto R Shimamura S Hiraga S Nakayama N Nakamura T Yukawa K Hachinohe M Matsumoto H Komatsu S Cytosolic ascorbate peroxidase 2 (cAPX 2) is involved in the soybean response to flooding Phytochem 2008 69 1295 1303 10.1016/j.phytochem.2008.01.007 18308350

[B15] Xu C Sullivan JH Garrett WM Caperna TJ Natarajan S Impact of solar Ultraviolet-B on the proteome in soybean lines differing in flavonoid contents Phytochem 2008 69 138 148 10.1016/j.phytochem.2007.06.010 17645898

[B16] Tanou G Job C Rajjou L Arc E Belghazi M Diamantidis G Molassiotis A Job D Proteomics reveals the overlapping roles of hydrogen peroxide and nitric oxide in the acclimation of citrus plants to salinity Plant J 2009 60 795 804 10.1111/j.1365-313X.2009.04000.x 19682288

[B17] Wang X Fan P Song H Chen X Li X Li Y Comparative proteomic analysis of differentially expressed proteins in shoots of *Salicornia europaea *under different salinity J Proteome Res 2009 8 3331 3345 10.1021/pr801083a 19445527

[B18] Tada Y Kashimura T Proteomic analysis of salt-responsive proteins in the mangrove plant, *Bruguiera gymnorhiza* Plant Cell Physiol 2009 50 439 446 10.1093/pcp/pcp002 19131358

[B19] Askari H Edqvist J Hajheidari M Kafi M Salekdeh GH Effects of salinity levels on proteome of *Suaeda aegyptiaca *leaves Proteomics 2006 6 2542 2554 10.1002/pmic.200500328 16612795

[B20] Salekdeh GH Siopongco J Wade LJ Ghareyazie B Bennett J A proteomic approach to analyzing drought- and salt-responsiveness in rice Field Crops Res 2002 76 199 219 10.1016/S0378-4290(02)00040-0

[B21] Umezawa T Shimuzu K Kato M Ueda T Enhancement of salt tolerance in soybean with NaCl pretreatment Physiol Plant 2000 110 59 63 10.1034/j.1399-3054.2000.110108.x

[B22] German MA Asher I Petreikov M Dai N Schaffer AA Granot D Cloning, expression and characterization of *LeFRK3*, the fourth tomato (*Lycopersicon esculentum *Mill.) gene encoding fructokinase Plant Sci 2004 166 285 291 10.1016/j.plantsci.2003.09.017

[B23] Mason HS Guerrero FD Boyer JS Mullet JE Proteins homologous to leaf glycoproteins are abundant in stems of darkgrown soybean seedlings. Analysis of proteins and cDNAs Plant Mol Biol 1988 11 845 856 10.1007/BF00019524 24272634

[B24] Staswick PE Soybean vegetative storage protein structure and gene expression Plant Physiol 1988 87 250 254 10.1104/pp.87.1.250 16666113 PMC1054734

[B25] Menegazzi P Guzzo F Baldan B Mariani P Treves S Purification of calreticulin-like protein(s) from spinach leaves Biochem Biophys Res Commun 1993 190 1130 1135 10.1006/bbrc.1993.1167 8439313

[B26] Wang W Vinocur B Shoseyov O Altman A Role of plant heat-shock proteins and molecular chaperones in the abiotic stress response Trends Plant Sci 2004 9 244 252 10.1016/j.tplants.2004.03.006 15130550

[B27] Zang X Komatsu S A proteomics approach for identifying osmotic-stress-related proteins in rice Phytochem 2007 68 426 437 10.1016/j.phytochem.2006.11.005 17169384

[B28] Jordan DB Chollet R Inhibition of ribulose bisphosphate carboxylase by substrate ribulose 1,5-bisphosphate J Biol Chem 1983 258 13752 13758 6417133

[B29] Rokka A Zhang L Aro EM Rubisco activase: an enzyme with a temperature dependent dual function? Plant J 2001 25 463 471 10.1046/j.1365-313x.2001.00981.x 11260502

[B30] Bertsch U Soll J Seetharam R Viitanen PV Identification, characterization, and DNA sequence of a functional "double" groES-like chaperonin from chloroplasts of higher plants Proc Natl Acad Sci USA 1992 89 8696 8700 10.1073/pnas.89.18.8696 1356267 PMC49987

[B31] Horvath I Multhoff G Sonnleitner A Vigh L Membrane-associated stress proteins: more than simply chaperones Biochim Biophys Acta 2008 1778 1653 1664 10.1016/j.bbamem.2008.02.012 18371297

[B32] Kotusov VV Kukhanova MK Krayevsky AA Gottikh BP Catalysis of the peptide bond formation by 50S subunits of *E. Coli *ribosomes with N-(formil) methionine ester of adenylic acid as peptide donor Mol Biol Rep 1976 3 151 156 10.1007/BF00423229 796686

[B33] Liu B Cyr RJ Palevitza BA A Kinesin-like Protein, KatAp, in the Cells of Arabidopsis and Other Plants Plant Cell 1996 8 119 132 10.1105/tpc.8.1.119 8597656 PMC161086

[B34] Oppenheimer DG Pollock MA Vacik J Szymanski DB Ericson B Feldmann K Mark D Essential role of a kinesin-like protein in Arabidopsis trichome morphogenesis Proc Natl Acad Sci USA 1997 94 6261 6266 10.1073/pnas.94.12.6261 9177205 PMC21037

[B35] Walker JE Arizmendi JM Dupuis A Fearnley IM Finel M Medd SM Pilkington SJ Runswick MJ Skehel JM Sequences of 20 subunits of NADH:ubiquinone oxidoreductase from bovine heart mitochondria. Application of a novel strategy for sequencing proteins using the polymerase chain reaction J Mol Biol 1992 226 1051 1072 10.1016/0022-2836(92)91052-Q 1518044

[B36] Trumper S Follmann H Haberlein I A novel dehydroascorbate reductase from spinach chloroplasts homologous to plant trypsin FEBS Lett 1994 352 159 162 10.1016/0014-5793(94)00947-3 7925967

[B37] Domash VI Sharpio TP Zabreiko SA Sosnovskaya TF Proteolytic enzymes and trypsin inhibitors of higher plants under stress conditions Rus J Bio Chem 2008 34 318 322 10.1134/S1068162008030114 18672684

[B38] Liao CT Lin CH Physiological adaptation of crop plants to flooding stress Proc Natl Sci Counc 2001 25 148 157 11480770

[B39] Cantero A Barthakur S Bushart TJ Chou S Morgan RO Fernandez MP Clark GB Roux SJ Expression profiling of the Arabidopsisannexin gene family during germination, de-etiolation and abiotic stress Plant Physiol Biochem 2006 44 13 24 10.1016/j.plaphy.2006.02.002 16531057

[B40] Gerke V Moss SE Annexins: from structure to function Physiol Rev 2001 82 331 271 10.1152/physrev.00030.2001 11917092

[B41] Schlomann M Schmidt E Knackmuss HJ Different types of dienelactone hydrolase in 4-fluorobenzoate utilizing bacteria J Bacter 1990 179 5112 5118 10.1128/jb.172.9.5112-5118.1990 PMC213169 2394679

[B42] Blasco R Wittich RM Mallavarapu M Timmis KN Pieper DH From xenobiotic to antibiotic, formation of protoanemonin from 4-Chlorocatechol by enzymes of the 3-Oxoadipate pathway J Biol Chem 1995 270 9229 9235 10.1074/jbc.270.49.29229 7493952

[B43] Grimmig B Matern U Structure of the parsley caffeoyl-CoA *O*-methyltransferase gene, harbouring a novel elicitor responsive *cis*-acting element Plant Mol Biol 1997 33 323 341 10.1023/A:1005780529457 9037150

[B44] Oommen A Dixon RA Paiva NL The elicitor-inducible alfalfa isoflavone reductase promoter confers different patterns of developmental expression in homologous and heterologous transgenic plants Plant Cell 1994 6 1789 1803 10.1105/tpc.6.12.1789 7866024 PMC160562

[B45] Mayer AM Polyphenol oxidases in plants and fungi: Going places? A review Phytochem 2006 67 2318 2331 10.1016/j.phytochem.2006.08.006 16973188

[B46] Frenkel C Klein I Dilley DR Protein synthesis in relation to ripening of pome fruits Plant Physiol 1968 13 1146 53 10.1104/pp.43.7.1146 PMC1086987 16656897

[B47] Kevany BM Tieman DM Taylor MG Cin VD Klee HJ Ethylene receptor degradation controls the timing of ripening in tomato fruit Plant J 2007 51 458 467 10.1111/j.1365-313X.2007.03170.x 17655616

[B48] Jeong MJ Park SC Byun MO Improvement of salt tolerance in transgenic potato plants by glyceraldehyde-3 phosphate dehydrogenase gene transfer Mol Cell 2001 12 185 189 11710519

[B49] Holmberg N Bulow L Improving stress tolerance in plants by gene transfer Trends Plant Sci 1998 3 61 66 10.1016/S1360-1385(97)01163-1

[B50] Livak KJ Schmittgen TD Analysis of relative gene expression data using real-time quqntitative PCR and the 2^-ΔΔ*C*T ^Method Methods 2001 25 402 408 10.1006/meth.2001.1262 11846609

[B51] Rozen S Skaletsky H Primer3 on the WWW for general users and for biologist programmers Meth Mol Biol 2000 132 365 386 10.1385/1-59259-192-2:365 10547847

