# Supplementary figures and images for: Proteome analysis of soybean leaves, hypocotyls and roots under salt stress
Source: Proteome Sci. 2010 Mar 29;8:19. doi: 10.1186/1477-5956-8-19 (PMC2859372; doi:10.1186/1477-5956-8-19)

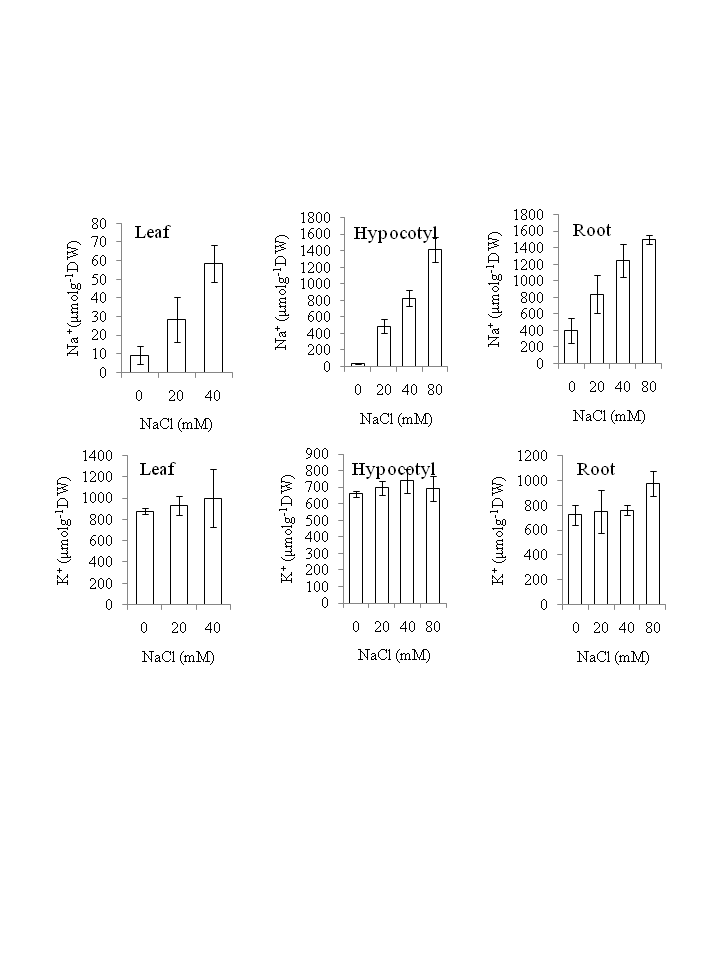

Supplement: Additional file 1 — Na and K contents in the leaf, hypocotyl and root of soybeans treated with NaCl. Soybeans were spwn on the sand and treated with 0, 20, 40 or 80 mM NaCl. They were grown for 2 weeks, and Na and K contents of leaf, hypocotyls and root were measured. Ten plants in each treatment were used. The experiments were repeated 3 times and the results show the average ± SE. [file 1477-5956-8-19-S1.TIFF]
