# Supplementary material for: Proteome analysis of soybean leaves, hypocotyls and roots under salt stress
Source: Proteome Sci. 2010 Mar 29;8:19. doi: 10.1186/1477-5956-8-19 (PMC2859372; doi:10.1186/1477-5956-8-19)
Supplement: Additional file 5 — Salt stress responsive proteins in leaves of soybean seedlings. a) Spot No, Spot number; b) The sequence shown is the N-terminal amino acid sequence determined by protein sequencing; c) Accession No, Accession number; d) Exp. Mr/pI shows experimental molecular weight and isoelectric point; e) Theor. Mr/pI shows theoretical molecular weight and pH isoelectric; f) PM, Number of matched peptides; g) SC, Sequence coverage by peptide mass fingerprinting using MALDI-TOF MS; h) U & D, up-regulated and down-regulated spots based on significant (p < 0.05) differences between control and NaCl treatments; i) CV ± SE, Spot volume of control ± standard error; j) TV ± SE, Spot volume of treatment ± standard error; k) T/C ratio, Treatment spot volume/control spot volume ratio; l) Category shows functional classification; m) ND, Not determined; M, metabolism; P, photosynthesis; D, defence; CT, cell transfer; PD, protein destination and storage; PS, protein synthesis; T, transcription. [file 1477-5956-8-19-S5.DOC]

| Spot No^a)^ | Amino acid sequence ^b)^ | Homologous protein | Accession  No ^c)^ | Exp.  Mr/ pI ^d)^ | Theor.  Mr/ pI ^e)^ | Identity  % | Score | PM ^f)^ | SC  (%) ^g)^ | U & D ^h)^ | CV±SE ^i)^ | TV±SE ^j)^ | T/C ratio ^k)^ | Category ^l)^ |
| --- | --- | --- | --- | --- | --- | --- | --- | --- | --- | --- | --- | --- | --- | --- |
| L01 | NVFFEERFDD | Calreticulin-1 | A0A762 | 68/4.3 | 48/4.4 | 100 | -- | -- | -- | U | 65.13 ± 6.85 | 255.17 ± 16.13 | 3.91 | D |
| L02 | blocked (MS) | ATP synthase CF1 beta subunit | Gm0262x00070  (YP_538748) | 60/5.3 | 53/5.3 | 100 | 148 | 11 | 35 | U | 105.30 ± 7.26 | 147.94 ± 5.95 | 1.40 | M |
| L03 | blocked (MS) | RuBisCO large subunit-binding protein subunit alpha (60 kDa chaperonin subunit alpha) | P08824 | 52/4.7 | 52/4.8 |  | -- | -- | -- | D | 74.89 ± 12.31 | 54.81 ±14.02 | 0.73 | P |
| L04 | blocked (MS) | Glyceraldehyde-3-phosphate dehydrogenase | Gm0047x00024  (ABC75834) | 34/6.4 | 37/6.7 | 99 | 60 | 5 | 17 | D | 71.10 ± 5.14 | 43.43 ± 3.88 | 0.61 | M |
| L05 | blocked (MS) | RuBisCO activase | Gm0081x00193.1  (O98997) | 43/4.8 | 48/7.6 | 89 | 98 | 9 | 18 | D | 121.00 ± 3.13 | 93.00 ± 5.30 | 0.76 | M |
| L06 | EGVPKRLTFD | Oxygen-evolving enhancer protein 1 | Q40459 | 31/4.9 | 35/5.9 | 100 | -- | -- | -- | U | 74.19 ± 6.65 | 94.60 ± 4.75 | 1.27 | P |
| L07 | EGVPKXLTFD | Oxygen-evolving enhancer protein 1 | Q40459 | 31/4.8 | 35/5.9 | 90 | -- | -- | -- | U | 24.93 ± 2.03 | 36.40 ± 11.28 | 1.45 | P |
| L08 | blocked (MS) | ND^m)^ | -- | 23/4.7 | -- |  | -- | -- | -- | D | 155.89 ± 20.19 | 93.74 ± 11.79 | 0.60 | -- |
| L09 | SSYADELVKT | Fructose-bisphosphate aldolase, chloroplast | P16096 | 36/5.9 | 42/6.9 | 100 | -- | -- | -- | D | 67.82 ± 6.34 | 38.53 ± 13.14 | 0.56 | M |
| L10 | ATVVA | 20 kDa chaperonin | P31233 | 25/5.7 | 22/10 | 100 | -- | -- | -- | U | 33.72 ±  10.14 | 78.19 ± 5.99 | 2.31 | PD |
| L11 | AEPEPVVDK | Protease inhibitor precursor | Q8RVX2 | 24/4.1 | 24/5.8 | 100 | -- | -- | -- | U | 53.97 ± 9.00 | 57.68 ± 20.07 | 1.06 | D |
| L12 | AVEAPEKITK | 50S ribosomal protein L12-3, chloroplast precursor | P36212 | 19/4.3 | 20/5.5 | 90 | -- | -- | -- | D | 197.65 ± 23.65 | 126.91 ± 20.99 | 0.64 | PS |
| L13 | blocked (MS) | RuBisCO large subunit | gi|21633955  (AAM55927) | 18/4.8 | 51/6.5 | 100 | 82 | 6 | 13 | D | 85.89 ± 13.27 | 66.82 ± 11.27 | 0.77 | P |
| L14 | TLSYLPDLDD | RuBisCO small subunit | Q42823 | 17/6.1 | 20/8.8 | 100 | -- | -- | -- | U | 55.50 ± 9.96 | 91.04 ± 7.88 | 1.64 | P |
| L15 | blocked (MS) | ND | -- | 59/4.9 | -- |  | -- | -- | -- | U | 27.61 ± 3.17 | 43.66 ± 15.81 | 1.58 | -- |
| L16 | blocked (MS) | ND | -- | 24/6.5 | -- |  | -- | -- | -- | U | 59.18 ± 27.24 | 77.52 ± 10.90 | 1.30 | -- |
| L17 | IAALKAPGF | RuBisCO large subunit-binding protein subunit beta (60 kDa chaperonin subunit beta) | P21241 | 29/5.2 | 62/6.6 | 100 | -- | -- | -- | U | 15.56 ± 2.48 | 34.24 ± 7.88 | 2.20 | P |
| L18 | KALXXSGGDH | RuBisCO large subunit | P30828 | 18/5.0 | 28/8.8 | 80 | -- | -- | -- | D | 93.23 ± 19.57 | 64.74 ± 26.52 | 0.69 | P |
| L19 | ERSSEVKXAS | Stem 31 kDa glycoprotein precursor | P10743 | 19/4.9 | 29/6.7 | 90 | -- | -- | -- | U | 65.61 ± 21.13 | 90.19 ± 14.33 | 1.37 | PD |
