# Supplementary material for: Proteome analysis of soybean leaves, hypocotyls and roots under salt stress
Source: Proteome Sci. 2010 Mar 29;8:19. doi: 10.1186/1477-5956-8-19 (PMC2859372; doi:10.1186/1477-5956-8-19)
Supplement: Additional file 6 — Salt stress responsive proteins in hypocotyls of soybean seedlings. a) Spot No, Spot number; b) The sequence shown is the N-terminal amino acid sequence determined by protein sequencing; c) Accession No, Accession number; d) Exp. Mr/pI shows experimental molecular weight and isoelectric point; e) Theor. Mr/pI shows theoretical molecular weight and pH isoelectric; f) PM, Number of matched peptides; g) SC, Sequence coverage by peptide mass fingerprinting using MALDI-TOF MS; h) U & D, up-regulated and down-regulated spots based on significant (p < 0.05) differences between control and NaCl treatments; i) CV ± SE, Spot volume of control ± standard error; j) TV ± SE, Spot volume of treatment ± standard error; k) T/C ratio, Treatment spot volume/control spot volume ratio; l) Category shows functional classification; m) ND, Not determined; M, metabolism; P, photosynthesis; D, defence; CT, cell transfer; PD, protein destination and storage; PS, protein synthesis; T, transcription. [file 1477-5956-8-19-S6.DOC]

| Spot No^a)^ | Amino acid sequence ^b)^ | Homologous protein | Accession  No ^c)^ | Exp.  Mr/ pI^d)^ | Theor.  Mr/ pI^e)^ | Identity  % | Score | PM ^f)^ | SC  (%)^g)^ | U & D ^h)^ | CV ± SE ^i)^ | TV ± SE ^j)^ | T/C ratio ^k)^ | Category^i^ |
| --- | --- | --- | --- | --- | --- | --- | --- | --- | --- | --- | --- | --- | --- | --- |
| H01 | -- | Kinesin motor protein | Gm0210x00045  (NP_850281) | 33/4 | 117/5.7 | 58 | 62 | 6 | 10 | U | 31.75 ± 3.74 | 47.65 ± 1.13 | 1.50 | CT |
| H02 | -- | Not hit | -- | 66/4.7 | -- | -- | -- | -- | -- | D | 74.87 ± 14.43 | 29.84 ± 4.58 | 0.39 | -- |
| H03 | AEPEPVV | NADH dehydrogenase 1 beta subcomplex subunit 8 | Q02372 | 26/4.7 | 22/5.7 | 100 | -- | -- | -- | D | 32.11 ± 5.15 | 5.95 ± 3.72 | 0.18 | M |
| H04 | DFVLDNEG | Trypsin inhibitor B | P01071 | 22/4.7 | 20/4.7 | 100 | -- | -- | -- | U | 36.11 ± 3.46 | 87.78 ± 2.83 | 2.43 | D |
| H05 | -- | Not hit | -- | 38/4.8 | -- | -- | -- | -- | -- | U | 23.18 ± 8.51 | 32.07 ± 3.28 | 1.38 | -- |
| H06 | -- | Methionine synthase | Gm0109x00038  (AAQ08403) | 77/6.8 | 84/5.9 | 98 | 70 | 9 | 17 | D | 93.35 ± 15.44 | 61.30 ± 4.24 | 0.65 | M |
| H07 | -- | Not hit | -- | 31/5.1 | -- | -- | -- | -- | -- | U | 27.68 ± 10.29 | 37.35 ± 3.40 | 1.34 | -- |
| H08 | -- | Transketolase | Gm0199x00050  (Q43848) | 76/6.8 | 81/6 | 82 | 67 | 8 | 13 | D | 74.00 ± 8.92 | 55.87 ± 6.26 | 0.75 | M |
| H09 | -- | Putative fructokinase 2 | Gm0134x00137  (AAQ10000) | 40/5.2 | 35/5.2 | 85 | 82 | 8 | 23 | D | 34.13 ± 4.04 | 33.84 ± 4.63 | 0.99 | M |
| H10 | DVEQVV | DNA-directed RNA polymerase gamma chain | P42074 | 22/5.1 | 23/6.5 | 83 | -- | -- | -- | U | 15.93 ± 5.71 | 22.79 ± 2.24 | 1.43 | T |
| H11 | ATVVAPKYTA | Acetoacetyl-CoA reductase | P50203 | 31/5.2 | 27/5.7 | 88 | -- | -- | -- | U | 22.05 ± 9.53 | 26.11 ± 12.51 | 1.18 | M |
| H12 | blocked (MS) | ND^m)^ | -- | 34/5.3 | -- | -- | -- | -- | -- | D | 75.80 ± 6.27 | 44.85 ± 2.66 | 0.59 | -- |
| H13 | -- | Stem 31 kDa glycoprotein precursor | Gm0113x00272.1  (P10743) | 33/5.3 | 29/6.7 | 100 | 79 | 5 | 24 | U | 87.16 ± 6.55 | 97.81 ± 5.51 | 1.12 | PD |
| H14 | AGTGKFFVGE | Triosephosphate isomerase, chloroplast precursor | Q9M4S8 | 31/5.4 | 33/7.7 | 100 | -- | -- | -- | D | 46.57 ± 10.20 | 38.19 ± 4.43 | 0.82 | M |
| H15 | GXVKIGIN | Glyceraldehyde-3-phosphate dehydrogenase | P08735 | 45/8.7 | 37/6.5 | 75 | -- | -- | -- | D | 62.02 ± 5.90 | 55.02 ± 6.97 | 0.88 | M |
| H16 | -- | Acid phosphatase | Gm0146x00211.2  (CAA11075) | 31/5.8 | 30/6.9 | 64 | 61 | 5 | 26 | D | 76.42 ± 9.31 | 55.38 ± 3.04 | 0.72 | M |
| H17 | -- | Not hit | -- | 37/6 | -- | -- | -- | -- | -- | U | 12.31 ± 3.60 | 16.20 ± 0.68 | 1.31 | -- |
| H18 | -- | Alcohol dehydrogenase Adh-1 | AAC62469 | 46/6.8 | 40/6.1 | 97 | 64 | 7 | 20 | U | 58.21 ± 6.60 | 93.17 ± 8.89 | 1.60 | M |
| H19 | -- | Not hit | -- | 17/7 | -- | -- | -- | -- | -- | U | 33.53 ± 2.39 | 50.96 ± 4.20 | 1.51 | -- |
| H20 | -- | Not hit | -- | 44/9 | -- | -- | -- | -- | -- | D | 85.83 ± 15.60 | 66.80 ± 11.31 | 0.77 | -- |
| H21 | -- | Annexin | Gm0100x00015  (AAB67993) | 37/8 | 35/6.4 | 81 | 86 | 8 | 25 | U | 32.14 ± 8.12 | 63.85 ± 7.19 | 1.98 | D |
| H22 | blocked (MS) | ND | -- | 49/8.5 | -- | -- | -- | -- | -- | U | 18.16 ± 8.59 | 44.43 ± 6.35 | 2.44 | -- |
