# Supplementary material for: Proteome analysis of soybean leaves, hypocotyls and roots under salt stress
Source: Proteome Sci. 2010 Mar 29;8:19. doi: 10.1186/1477-5956-8-19 (PMC2859372; doi:10.1186/1477-5956-8-19)
Supplement: Additional file 7 — Salt stress responsive proteins in roots of soybean seedlings. a) Spot No, Spot number; b) The sequence shown is the N-terminal amino acid sequence determined by protein sequencing; c) Accession No, Accession number; d) Exp. Mr/pI shows experimental molecular weight and isoelectric point; e) Theor. Mr/pI shows theoretical molecular weight and pH isoelectric; f) PM, Number of matched peptides; g) SC, Sequence coverage by peptide mass fingerprinting using MALDI-TOF MS; h) U & D, up-regulated and down-regulated spots based on significant (p < 0.05) differences between control and NaCl treatments; i) CV ± SE, Spot volume of control ± standard error; j) TV ± SE, Spot volume of treatment ± standard error; k) T/C ratio, Treatment spot volume/control spot volume ratio; l) Category shows functional classification; m) ND, Not determined; M, metabolism; P, photosynthesis; D, defence; CT, cell transfer; PD, protein destination and storage; PS, protein synthesis; T, transcription. [file 1477-5956-8-19-S7.DOC]

| Spot No^a)^ | Amino acid sequence ^b)^ | Homologous protein | Accession  No ^c)^ | Exp.  Mr/ pI^d)^ | Theor.  Mr/ pI^e)^ | Identity  % | Score | PM ^f)^ | SC  (%)^g)^ | U & D ^h)^ | CV ± SE ^i)^ | TV ± SE ^j)^ | T/C ratio ^k)^ | Category ^l)^ |
| --- | --- | --- | --- | --- | --- | --- | --- | --- | --- | --- | --- | --- | --- | --- |
| R01 | -- | Putative fructokinase 2 | Gm0137x00074  (AAQ10000) | 40/4.7 | 35/5.2 | 88 | 107 | 8 | 25 | D | 191.83 ± 30.71 | 47.30 ± 31.52 | 0.24 | M |
| R02 | -- | Dienelactone hydrolase family protein | Gm0009x00009.1  (NP_180811) | 32/52 | 26/5.3 | 83 | 81 | 5 | 31 | D | 87.60 ± 26.59 | 8.30 ± 1.26 | 0.09 | M |
| R03 | blocked (MS) | ND^m)^ | -- | 18/5.2 | -- | -- | -- | -- | -- | U | 242.73 ± 17.43 | 349.0 7 ± 11.86 | 1.43 | -- |
| R04 | blocked (MS) | Not hit | -- | 30/5.3 | -- | -- | -- | -- | -- | D | 139.00 ± 10.17 | 94.07 ± 11.34 | 0.67 | -- |
| R05 | blocked (MS) | Not hit | -- | 21/5.4 | -- | -- | -- | -- | -- | U | 5.49 ± 3.02 | 48.29 ± 9.35 | 8.79 | -- |
| R06 | -- | Caffeoyl-CoA-O-methyltransferase | Gm0102x00056  (ABF74683) | 34/5.4 | 28/5.3 | 94 | 82 | 6 | 33 | D | 79.41 ± 9.58 | 24.85 ± 9.67 | 0.31 | M |
| R07 | -- | NADPH:isoflavone reductase | Gm0013x00297  (CAA06027) | 40/5.3 | 36/5.3 | 99 | 70 | 6 | 20 | D | 252.02 ± 42.12 | 57.37 ± 33.58 | 0.22 | M |
| R08 | blocked (MS) | Not hit | -- | 39/5.6 | -- | -- | -- | -- | -- | D | 217.69 ± 28.78 | 54.63 ± 33.33 | 0.25 | -- |
| R09 | -- | Putative cinnamyl alcohol dehydrogenase | Gm0098x00216.2  (AAC06319) | 37/6.1 | 35/6.6 | 73 | 64 | 5 | 19 | U | 183.62 ± 12.87 | 285.15 ± 18.75 | 1.55 | D |
| R10 | -- | Putative quinone oxidoreductase | Gm0030x00514.1  (CAD31838) | 27/7 | 22/6.5 | 92 | 99 | 6 | 39 | D | 486.27 ± 95.08 | 213.41 ± 6.86 | 0.43 | M |
| R11 | -- | Stem 31 kDa glycoprotein precursor | gi|134146  (P10743) | 33/6.5 | 29/6.7 | 100 | 104 | 7 | 27 | U | 280.26 ± 22.76 | 613.79 ± 123.83 | 2.19 | PD |
| R12 | blocked (MS) | Not hit | -- | 14/9 | -- | -- | -- | -- | -- | U | 5.00 ± 1.31 | 144.00 ± 35.63 | 28.79 | -- |
| R13 | -- | Ripening related protein | Gm0008x00017  (AAD50376) | 18/8.2 | 18/5.4 | 79 | 84 | 6 | 50 | U | 33.20 ± 16.40 | 305.66 ± 93.78 | 9.20 | D |
| R14 | blocked (MS) | Not hit | -- | 85/5 | -- | -- | -- | -- | -- | U | 562.16 ± 79.34 | 651.65 ± 110.23 | 1.15 | -- |

a) Spot No, Spot number; b) The sequence shown is the N-terminal amino acid sequence determined by protein sequencing; c) Accession No, Accession number; d) Exp. Mr/pI shows experimental molecular weight and isoelectric point; e) Theor. Mr/pI shows theoretical molecular weight and pH isoelectric; f) PM, Number of matched peptides; g) SC, Sequence coverage by peptide mass fingerprinting using MALDI-TOF MS; h) U & D, up-regulated and down-regulated spots based on significant (*p* < 0.05) differences between control and NaCl treatments; i) CV ± SE, Spot volume of control ± standard error; j) TV ± SE, Spot volume of treatment ± standard error; k) T/C ratio, Treatment spot volume/ control spot volume ratio; l) Category shows functional classification; m) ND, Not determined; M, metabolism; P, photosynthesis; D, defence; CT, cell transfer; PD, protein destination and storage; PS, protein synthesis; T, transcription.
